# College Notes

**Published:** 1893-09

**Authors:** 


### College Notes.

The Medical Department of Niagara University opens its doors Monday, September 25, 1893.

The Medical Department of the University of Buffalo commences its forty-eighth regular session on Monday, September 25, 1893.

The following changes have been made in the faculty of the Medcal Department, University of Buffalo: Dr. S. Y. Howell has resigned, and Dr. W. H. Bergtold has been appointed Professor of Pathology; Dr. J. B. Andrews has been made Emeritus Professor of Insanity; Dr. A. W. Hurd has been appointed Lecturer on Insanity.

The annual announcements of the Buffalo medical schools are more tastefully and elaborately gotten up than any that have been received in many years. They contain a lot of information, are handsomely illustrated, and reflect credit on their respective schools. Readers of the Journal desiring copies can obtain them by addressing Dr. A. A. Hubbell or Dr. John Parmenter.

